# A Pilot Study of Chemoradiotherapy With Weekly Docetaxel for Thoracic Esophageal Carcinoma With T4 and/or M1 Lymph Node Metastasis

**DOI:** 10.4021/wjon407w

**Published:** 2011-10-28

**Authors:** Isamu Makino, Itasu Ninomiya, Koichi Okamoto, Jun Kinoshita, Hironori Hayashi, Keishi Nakamura, Katsunobu Oyama, Hisatoshi Nakagawara, Hideto Fujita, Hidehiro Tajima, Hiroyuki Takamura, Hirohisa Kitagawa, Sachio Fushida, Takashi Tani, Takashi Fujimura, Tetsuo Ohta, Tsuyoshi Takanaka

**Affiliations:** a Department of Gastroenterologic Surgery, Graduate School of Medical Science, Kanazawa University, 13-1 Takaramachi, Kanazawa, Ishikawa 920-8641, Japan; b Department of Radiology, Graduate School of Medical Science, Kanazawa University, 13-1 Takaramachi, Kanazawa, Ishikawa 920-8641, Japan

**Keywords:** Esophageal cancer, Squamous cell carcinoma, Docetaxel, Chemoradiotherapy

## Abstract

**Background:**

Patients with unresectable or inoperable esophageal carcinoma are usually treated with definitive chemoradiotherapy. The present standard regimen is radiation with concurrent chemotherapy with cisplatin and fluorouracil. However, significant toxicities have been observed. The efficacy and safety of concurrent chemoradiotherapy with weekly docetaxel for head-and-neck squamous cell carcinoma and non-small cell lung cancer have already been recognized. We conducted a pilot study of definitive chemoradiotherapy with weekly docetaxel for advanced esophageal carcinoma.

**Methods:**

Nine patients with advanced thoracic esophageal squamous cell carcinoma having a T4 tumor and/or distant lymph node metastasis (M1 LYM) were enrolled. Docetaxel was administered concurrently with 60 Gy of radiation by drip infusion at a dose of 10 mg/m^2^ for an hour once per week and 6 times in total.

**Results:**

All 9 patients completed the treatment schedule without any suspension. Grade 3 or higher hematological and biochemical toxicities did not occur. Two patients achieved complete response, and 4 achieved partial response. The response rate was 67%. The median survival time was 16.2 months and the 2-year survival rate was 38.9%.

**Conclusions:**

Concurrent chemoradiotherapy with weekly low dose docetaxel is a safe and effective treatment regimen for esophageal squamous cell carcinoma. We expect that this protocol of chemoradiotherapy may be one of the choices of treatment substituting the regimen with cisplatin and fluorouracil, particularly for the patients for whom chemotherapy with cisplatin and fluorouracil is considered inappropriate because of concomitant renal dysfunction or prior failure of systemic chemotherapy with cisplatin and fluorouracil.

## Introduction

Carcinoma of the esophagus is a highly malignant disease and the treatment outcome has been poor. Patients with unresectable or inoperable disease are usually treated with definitive chemoradiotherapy. Based on the results of the Radiation Therapy Oncology Group (RTOG) phase III intergroup trial RTOG 85-01, the present standard regimen is radiation with concurrent chemotherapy with cisplatin and fluorouracil [1, 2]. In Japan, 2 phase II studies of definitive chemoradiotherapy with cisplatin and fluorouracil for advanced thoracic esophageal squamous cell carcinoma (TESCC) with T4 tumor and/or distant lymph node metastasis (M1 LYM) were performed [3, 4]. The complete response (CR) rate of each study was 33% [3] and 15% [4], and the survival rate was 23% at 3 years [3], and 31.5% at 2 years [4], respectively. However, significant toxicities, particularly severe hematological toxicities associated with these treatment regimens, have been observed [3, 4].

The efficacy and safety of concurrent chemoradiotherapy with weekly docetaxel for head-and-neck squamous cell carcinoma (HNSCC) [5-8] and non-small cell lung cancer (NSCLC) [9-12] have already been recognized. According to Japanese reports [7], the recommended dose of docetaxel in the concurrent chemoradiotherapy for HNSCC was weekly administration of 10 mg/m^2^.

We conducted a pilot study of concurrent chemoradiotherapy with weekly docetaxel for TESCC with T4 and/or M1 LYM. We administered docetaxel according to the protocol described in Japanese reports of HNSCC [7]. Herein, we report the efficacy and safety of this regimen.

## Patients and Methods

### Patients

From October 2005 to March 2009, 9 patients with advanced TESCC having a T4 tumor and/or M1 LYM were enrolled in this study. The criteria for inclusion in this study were as follows: newly diagnosed and pathologically confirmed squamous cell carcinoma of the thoracic esophagus; T4 tumor and/or M1 LYM according to the TNM classification of the UICC International Union Against Cancer, 6^th^ edition; no evidence of esophagotracheal or esophagobronchial fistula and distant organ metastasis; performance status (PS) of 0-2 based on the classification criteria of the Eastern Cooperative Oncology Group; age 20-80 years; adequate bone marrow [white blood cell (WBC) count ≥ 3,000 /µl, neutrocyte count ≥ 1,500 /µl, hemoglobin (Hb) level ≥ 10.0 g/dl, platelet count ≥ 100,000 /µl], hepatic (total bilirubin < 1.5 mg/dl, alanine transaminase (ALT), aspartate transaminase (AST) < 3.0 × normal limit), pulmonary (PO_2_ ≥ 60 mmHg), and renal (serum creatinine < 2.0 mg/dl, creatinine clearance ≥ 50 ml/min) functions. Patients with other active synchronous carcinoma, concurrent uncontrolled medical illness, and prior chemotherapy and radiotherapy for any neoplasm were excluded. All patients provided written informed consent before enrollment in this study.

### Treatment schedule

**Figure 1:**
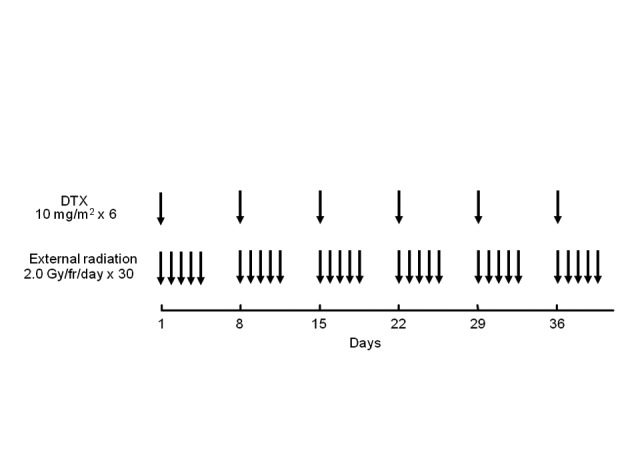
Treatment schedule of chemoradiotherapy with weekly docetaxel. DTX: docetaxel.

The treatment schedule is summarized in Fig. 1. Docetaxel was administered concurrently with radiotherapy by drip infusion at a dose of 10 mg/m^2^ for an hour once per week and 6 times in total. Radiation was administered via 10 MV X-ray (1 fraction per day and 5 fractions per week). When the tumor was located in the upper or middle third of the thoracic esophagus, the treatment volume included bilateral supraclavicular lymph nodes as well as the mediastinum in a T-shaped pattern. When the tumor was located in the lower third of the esophagus, the mediastinum and celiac axis lymph nodes were irradiated and the supraclavicular lymph nodes were excluded if the cervical nodes tested negative. Oblique fields were used to spare the spinal cord after 40 Gy of radiation was delivered by antero-posterior opposed pair portals. Weekly administration of docetaxel was skipped when the WBC, neutrocyte, or platelet count decreased to < 3,000 /µl, < 1,500 /µl, or < 75,000 /µl, respectively. Radiotherapy was suspended when the WBC, neutrocyte, or platelet count decreased to < 1,000 /µl, < 500 /µl, or < 50,000 /µl, and was resumed when the counts recovered to ≥ 1,000 /µl, ≥ 500 /µl, and ≥ 50,000 /µl, respectively.

### Evaluation of toxicity and response

Adverse reactions were evaluated according to the National Cancer Institute’s Common Terminology Criteria for Adverse Events (NCI-CTCAE), version 4.0. The tumor response associated with the treatment was evaluated according to the Response Evaluation Criteria in Solid Tumors (RECIST) [13]. The response in primary tumors, which were considered as non-target lesions, was examined by endoscopy and evaluated according to the Response Evaluation Criteria for Primary Lesion using Endoscopy defined in the Japanese Classification of Esophageal Cancer published by the Japanese Esophageal Society [14].

### Statistical analysis

Overall survival time was defined as the time from initiation of the treatment to the date of death or date of final follow-up examination. Survival rates were estimated by the Kaplan-Meier method. All statistical calculations were carried out using SPSS software version 11 (SPSS Inc; Chicago, IL).

## Results

### Patient characteristics

Between October 2005 and March 2009, 9 patients were enrolled in this study. The characteristics of these 9 patients are listed in Table 1. The median age was 66 years (range, 51 to 78 years). There were 4 patients with non-T4 M1 LYM, 4 patients with T4 M0, and 1 patient with T4 M1 LYM. Four patients had a T4 tumor that invaded into the tracheobronchial tree and 1 patient had a T4 tumor that invaded into both the tracheobronchial tree and thoracic aorta. Among the 5 patients with M1 LYM, 4 patients had cervical lymph node metastases, and 1 patient had both cervical and abdominal lymph node metastases.

**Table 1:** Patient characteristics

Characteristic	n = 9
Age (years)	
Median	66
Range	51-78
Gender	
Female	1
Male	8
Performance status	
0	7
1	2
UICC TNM stage	
non-T4 M1 LYM	4
T4 M0	4
T4 M1 LYM	1
Primary tumor site	
Upper thoracic esophagus	3
Middle thoracic esophagus	5
Lower thoracic esophagus	1
Site of M1 LYM disease	
Cervical node	4
Cervical and abdominal node	1

### Toxicity

All patients completed treatment with a total radiation dose of 60 Gy without any suspension. The most severe toxicities during the treatment and follow-up period are summarized in Table 2. Grade 3 or higher hematological and biochemical toxicities did not occur. The most frequent toxicities were anorexia and esophagitis. A patient with T4 disease developed treatment-related perforation of the esophageal wall and died because of esophago-mediastinal fistula 27 months after initiation of the treatment. There was 1 (11%) death related to the treatment.

**Table 2:** Summary of toxicity

	Grade (n = 9)					% ≥ Grade 3
	0	1	2	3	4	
Acute toxicity						
Hematological						
Leukopenia	8	0	1	0	0	0
Neutropenia	8	0	1	0	0	0
Anemia	4	3	1	0	0	0
Thrombocytopenia	8	0	1	0	0	0
Biochemical						
Bilirubin	9	0	0	0	0	0
AST	7	2	0	0	0	0
ALT	7	2	0	0	0	0
Creatinine	8	1	0	0	0	0
Non-hematological						
Anorexia	6	1	0	2	0	22
Nausea	9	0	0	0	0	0
Vomiting	8	0	1	0	0	0
Esophagitis	6	1	0	2	0	22
Esophageal bleeding	7	0	1	1	0	11
Dermatitis	7	1	1	0	0	0
Late toxicity						
Peumonitis	9	0	0	0	0	0
Pleural effusion	8	0	0	1	0	11
Pericardial effusion	9	0	0	0	0	0

### Response

Individual data of the treatment response and prognosis are listed in Table 3. Of the 9 patients, 2 (22%) achieved CR, and 4 (44%) achieved PR. The response rate was 67% (Table 4). The other 3 patients (33%) had progressive disease (PD). PD in these 3 patients developed because of progression of the primary lesion, development of lung metastases, and development of bone metastases, respectively. As far as the response of primary tumors were concerned, 4 patients (44%), including one with T4 disease, achieved CR.

**Table 3:** Treatment response and prognosis of 9 patients

Case No.	Age	Gender	UICC TNM	Tumor site	Tumor size (cm)	Overall response	Primary response	Prognosis
1	54	M	T4 M0	Middle	8	PD	PD	8 M died of disease
2	78	M	T4 M0	Upper	12	CR	CR	27 M died of esophageal perforation
3	60	M	T4 M0	Middle	15	PD	PD	2 M died of disease
4	51	M	T1 M1 LYM	Lower	5	PD	CR	36 M alive with disease progression
5	66	M	T1 M1 LYM	Middle	3	PR	CR	29 M died of disease
6	66	F	T3 M1 LYM	Middle	12	CR	CR	16 M died of disease
7	70	M	T3 M1 LYM	Middle	10	PR	IR/SD	5 M alive with disease progression
8	58	M	T4 M1 LYM	Upper	6	PR	IR/SD	9 M died of disease
9	70	M	T4 M0	Upper	13	PR	IR/SD	4 M died of disease

PD, progressive disease; CR, complete response; PR, partial response; IR, incomplete response; SD, stable disease.

**Table 4:** Summary of response and survival

Number of patients	9
Response rate	67% (CR 22%; PR 44%)
Median survival time	16.2 months (95% CI: 4.9-27.5)
2-year survival rate	38.9%

CR, complete response; PR, partial response; CI, confidence interval.

### Survival

**Figure 2:**
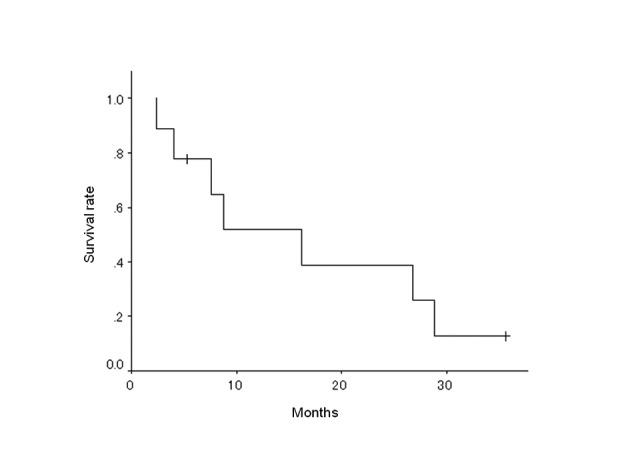
Overall survival of 9 patients.

The prognoses of the 9 patients are listed in Table 3, and the overall survival curves are shown in Fig. 2. The median survival time (MST) was 16.2 months (95% confidence interval: 4.9-27.5 months) and the 2-year survival rate was 38.9% (Table 4).

## Discussion

The present standard regimen of chemoradiotherapy for TESCC is radiation and concurrent chemotherapy with cisplatin and fluorouracil [1-4]. However, because other treatment regimens have not been established, there is no recommended choice of treatment for patients for whom chemotherapy with cisplatin and fluorouracil is considered inappropriate because of concomitant renal dysfunction or prior failure of systemic chemotherapy with cisplatin and fluorouracil. Moreover, significant toxicities, particularly severe hematological toxicities, associated with this treatment regimen, have been observed [1-4]. Other effective and reliable chemoradiotherapy regimens in addition to the one with cisplatin and fluorouracil are needed.

The concurrent use of taxanes (docetaxel and paclitaxel) with radiation has been employed for the treatment of patients with HNSCC [5-8] and NSCLC [9-12]. Many clinical and experimental studies have demonstrated the efficacy of concomitant administration of taxanes with radiation [15-22]. The efficacy of taxanes is based on not only their cytotoxicity but also their radio-sensitizing effect. Microtubule modification initiated by taxanes causes cells to accumulate in the radio-sensitive G2/M phase of the cell cycle. The radio-sensitizing effect of taxanes is thought to be derived mainly from this mechanism. Furthermore, in vitro and in vivo studies have demonstrated roles for the p53 pathways and apoptotic mechanism as contributing factors of the therapeutic benefits of combining taxanes with concurrent radiation.

Taxanes are usually administered every 3 weeks as a single cytotoxic agent; however, there are several reports showing that weekly administration of taxanes may be the most effective dosing regimen in combination with radiation [12, 23]. Most of regimens of docetaxel with concurrent radiotherapy consist of weekly administration of docetaxel at a dose of 10-30 mg/m^2^ [5-12]. The most appropriate dosage of docetaxel may be different among the types of tumor, range of irradiation fields, and ethnic origin of the patients. In this pilot study, we administered docetaxel according to the protocol described in Japanese reports of HNSCC [7], considering the racial identity and oncological similarity between TESCC and HNSCC.

In the present study, the overall CR and response rate were 22% and 67%, respectively. The median survival time was 16.2 months and the 2-year survival rate was 38.9%. These results are compatible to those in previous Japanese reports of concurrent chemoradiotherapy with cisplatin and fluorouracil for TESCC with T4 and/or M1 LYM [3, 4]. However, grade 3 or higher hematological toxicities did not occur in the present study, although these complications occurred in approximately 20%-30% of patients in the previous Japanese reports [3, 4]. In our study, all 9 patients completed the treatment schedule without any suspension. A critical adverse event occurred in 1 of the 9 patients (11%), who developed perforation of the esophageal wall and died because of esophago-mediastinal fistula. Although the present study has a limited number of patients, concurrent chemoradiotherapy with weekly low dose docetaxel might be a feasible and promising treatment regimen for TESCC.

We expect that this protocol of chemoradiotherapy may be one of the choices of treatment for TESCC, particularly for the patients for whom chemotherapy with cisplatin and fluorouracil is considered inappropriate because of concomitant renal dysfunction or prior failure of systemic chemotherapy with cisplatin and fluorouracil. Moreover, as one of the treatment options, we plan to combine this regimen with prior induction chemotherapy with cisplatin and fluorouracil to achieve a survival benefit for the patients with advanced TESCC. We are currently conducting a phase I/II study of chemoradiotherapy with docetaxel following induction chemotherapy with cisplatin and fluorouracil in order to determine the appropriate dosage for the weekly administration of docetaxel.
